# Spread of *Cryptococcus gattii* into Pacific Northwest Region of the United States

**DOI:** 10.3201/eid1508.081384

**Published:** 2009-08

**Authors:** Kausik Datta, Karen H. Bartlett, Rebecca Baer, Edmond Byrnes, Eleni Galanis, Joseph Heitman, Linda Hoang, Mira J. Leslie, Laura MacDougall, Shelley S. Magill, Muhammad G. Morshed, Kieren A. Marr, for the Cryptococcus gattii Working Group of the Pacific Northwest

**Affiliations:** Johns Hopkins University School of Medicine, Baltimore, Maryland, USA (K. Datta, K.A. Marr); University of British Columbia, Vancouver, British Columbia, Canada (K.H. Bartlett); Washington State Department of Health, Shoreline, Washington, USA (R. Baer); Duke University Medical Center, Durham, North Carolina, USA (E. Byrnes, J. Heitman); British Columbia Centre for Disease Control, Vancouver (E. Galanis, L. Hoang, L. MacDougall, M.G. Morshed); British Columbia Ministry of Agriculture and Lands, Abbotsford, British Columbia, Canada (M.J. Leslie); Centers for Disease Control and Prevention, Atlanta, Georgia, USA (S.S. Magill)

**Keywords:** Cryptococcus gattii, cryptococcosis, fungi, Vancouver Island, Pacific northwest, Canada, United States, synopsis

## Abstract

*Cryptococcus gattii* has emerged as a human and animal pathogen in the Pacific Northwest. First recognized on Vancouver Island, British Columbia, Canada, it now involves mainland British Columbia, and Washington and Oregon in the United States. In Canada, the incidence of disease has been one of the highest worldwide. In the United States, lack of cryptococcal species identification and case surveillance limit our knowledge of *C. gattii* epidemiology. Infections in the Pacific Northwest are caused by multiple genotypes, but the major strain is genetically novel and may have emerged recently in association with unique mating or environmental changes. *C. gattii* disease affects immunocompromised and immunocompetent persons, causing substantial illness and death. Successful management requires an aggressive medical and surgical approach and consideration of potentially variable antifungal drug susceptibilities. We summarize the study results of a group of investigators and review current knowledge with the goal of increasing awareness and highlighting areas where further knowledge is required.

## Abstract 2

This organism should be recognized as an emerging pathogen in the United States.

*Cryptococcus gattii* (formerly *C*. *neoformans* var. *gattii*) (*1*) is a basidiomycotic yeast acquired by inhalation. In a susceptible host, the disease (cryptococcosis), caused by *C*. *gattii* and the congeneric pathogen *C*. *neoformans*, usually results in pneumonia or dissemination to distant tissues, especially to the central nervous system. Some studies have shown that *C*. *gattii* appears to differ from other cryptococcal pathogens in phenotypic characteristics, natural habitat, epidemiology, clinical disease manifestations and response to antifungal drugs. We briefly summarize these features before discussing the emergence of *C*. *gattii* in the Pacific Northwest region of Canada and the United States.

Based on the distribution of specific antigenic determinants on the polysaccharide capsule, pathogenic cryptococci have been divided into capsular serotypes B and C (both *C*. *gattii*), A (*C*. *neoformans* var. *grubii*), D (*C*. *neoformans* var. *neoformans*), and the hybrid diploid AD (*1*). Clinical and environmental *C*. *gattii* isolates obtained from most parts of the world belong to serotype B (*2*), the serotype also responsible for cryptococcal disease in the Pacific Northwest. The global distribution of *C*. *gattii* serotype C appears relatively more geographically restricted (*2*–*4*).

*C*. *gattii* causes disease in healthy, immunocompetent persons and in persons with immunosuppressive conditions, including those with HIV infection, organ transplant recipients, and patients with hematologic malignancies (*2*,*3*,*5*,*6*). However, because clinical cryptococcal isolates are not routinely subtyped to the serotype or species level, the actual incidence of *C*. *gattii* infection in HIV-negative persons is not known. In a US population-based surveillance study, 2 of 27 cryptococcal isolates obtained from HIV-negative persons were *C*. *gattii* (*7*). Although *C*. *neoformans* serotype A has been the most common cause of human disease worldwide since the HIV pandemic began, *C*. *gattii* may have previously caused proportionally more disease. For example, in Thailand in the pre-AIDS era, *C*. *gattii* comprised 66% of cryptococcal isolates, which decreased to ≈4% during the AIDS epidemic (*8*).

In general, cryptococcal disease can occur in 1 of 2 ways: a primary disease progression in the context of immunosuppression, or reactivation of a subclinical, latent infection. Progression from infection or colonization to disease likely depends upon a complex interplay of factors associated with the host and the organism.

The natural history of *C*. *gattii* infection is not well understood, but some studies suggest subtle differences in disease caused by *C*. *gattii* and *C*. *neoformans*. *C*. *gattii* causes cryptococcomas in the lung and brain (often large, multifocal lesions) more commonly than *C*. *neoformans*, and *C*. *gattii* disease is more often associated with neurologic sequelae, frequently requiring aggressive neurosurgical management (*9*,*10*). Differences in clinical manifestations and outcome suggested by some studies may in part be explained by differences in host immune status (*9*), although not all studies have demonstrated these differences (*4*).

Some reports have observed variable-to-decreased in vitro antifungal drug susceptibilities in clinical and environmental *C*. *gattii* isolates (*11*,*12*); other studies have shown no differences (*4*). Clinical significance of in vitro susceptibility of cryptococcal isolates is unclear because testing methods are not standardized and MIC breakpoints have not been defined.

*C*. *gattii* is found typically in the tropics and subtropics (*2*). Accordingly, although detected in many regions, *C*. *gattii* has been found to be endemic to Australia and New Zealand, Papua New Guinea, South and Southeast Asia (Cambodia, Malaysia, Thailand, Vietnam, People’s Republic of China, Taiwan, Singapore, Nepal, and the Indian subcontinent), parts of Latin America (Argentina, Brazil, Colombia, Uruguay, Paraguay, Peru, and Venezuela), southern California, Mexico, Hawaii, Central and South Africa, and certain parts of Europe (Austria, Germany, France, Italy, Greece, and Spain) (*2**,**13*).

Our understanding of the epidemiology of cryptococcosis is limited by our currently used diagnostic methods. Diagnosis of cryptococcosis frequently relies on direct microscopy, culture of clinical samples, or detection of cryptococcal antigen in body fluids. However, the organism is not identified to species level in many clinical microbiology laboratories. One biochemical characteristic that distinguishes *C*. *gattii* from *C*. *neoformans* is its ability to produce blue coloration on l-canavanine-glycine-bromothymol blue (CGB) medium (*14*), although, occasionally, some isolates identified as *C*. *gattii* by molecular typing may be CGB negative (L. Hoang, unpub. data).

Molecular typing studies, which are essential in tracking *C*. *gattii* epidemiology, have used the techniques of PCR fingerprinting (*15*), restriction fragment length polymorphism (RFLP) analysis (*16*), intergenic spacer sequencing (*17*), amplified fragment length polymorphism analysis (*18*), and more recently, multilocus sequence typing (MLST) (*19*,*20*). Molecular typing has identified distinct haploid *C*. *gattii* lineages among clinical, veterinary, and environmental isolates, such as VGI, VGII, VGIII, and VGIV (*19*,*21*,*22*), which appear to have distinct biogeoclimatic distribution zones. For example, most clinical isolates from Australia and eucalyptus-associated *C*. *gattii* isolates belong to molecular type VGI; VGII isolates have been found in domestic animals in Australia and in Aboriginal persons in the Northern Territory (*2*). On Vancouver Island and mainland British Columbia, most human, veterinary, and environmental *C*. *gattii* isolates belong to the genotype VGII, with its 2 molecular subtypes VGIIa (predominant genotype, ≈90% of VGII) and VGIIb (≈10%), representing 2 independent clonal populations (*20*,*22*,*23*).

## Emergence on Vancouver Island and in Mainland British Columbia

Isolated cases of *C*. *gattii* disease were identified in the animal population of British Columbia in 2000 (*24*). *C*. *gattii* disease was recognized as an epidemic on Vancouver Island and lower mainland British Columbia (*23*,*25*) and retrospectively found to have been affecting residents and travelers to the island, as well as the domestic and wild animal population, since 1999 (*24*).

In 2003, cryptococcal infection became a reportable disease in British Columbia, which strengthened surveillance efforts. The British Columbia Centre for Disease Control reported a cumulative incidence of 218 *C*. *gattii* cases in humans during 1999–2007, with an average of 24.2 cases per year (*26*). The average annual incidence rate of *C*. *gattii* infection was 5.8 cases per million on mainland British Columbia and 25.1 cases per million on Vancouver Island; this rate is higher than those reported in other *C. gattii*–endemic areas of the world (*27*). During this same period, 19 deaths from *C*. *gattii* disease were documented, for a case-fatality rate of 8.7% (*26*). Of these 218 cases, 55% were in men; the mean age at diagnosis was 58.7 years. Possible risk factors included smoking, cancer, and HIV infection (*26*). Annual incidence, after reaching a plateau during 2000–2005, increased in 2006. Although most *C*. *gattii* cases have occurred among residents of Vancouver Island the numbers of cases have steadily increased among residents of lower mainland British Columbia, likely caused by an active expansion of the epidemic zone since 2004 from Vancouver Island to mainland British Columbia and the northwestern United States (Washington and Oregon) (*27*). The exact mode of transmission of *C*. *gattii* from Vancouver Island to lower mainland British Columbia is not known. However, studies of possible dispersal mechanisms have indicated an association of *C*. *gattii* cases with high-traffic locations, and evidence of anthropogenic dispersal through vehicle wheel wells, footwear, construction and forestry activity (leading to aerial dispersal), and water (*23*). A few domestic and international travelers to the disease-endemic zone on Vancouver Island have subsequently contracted cryptococcal disease caused by *C*. *gattii* VGIIa, the molecular subtype most commonly associated with the Vancouver Island emergence (*28*,*29*).

## Emergence in the Northwestern United States

Use of MLST has documented that a *C*. *gattii* strain genetically similar to the Vancouver Island VGIIa strain caused disease in a patient in Seattle in 1971 (strain NIH444, also known as ATCC32609 and CBS6956). Another similar isolate was found in the environment in San Francisco, California, in 1990 (strain CBS7750) (*20*,*22*). These findings indicate that this subtype of *C*. *gattii* was present in the environment (and may have been a cause of human disease) in this part of the United States for many years before recognition of the Vancouver Island emergence (*20*). In recent years, several cases of *C*. *gattii* disease have been recognized in Washington and Oregon.

In early 2006, the first US case of human infection by a *C*. *gattii* strain identical to the Vancouver Island VGIIa (as confirmed by MLST) was diagnosed in a resident of Orcas Island, Washington (*30*). From 2006 through July 2008, a total of 9 additional culture-confirmed cases were reported in Washington in residents of Whatcom, Island, King, and San Juan counties (R. Baer, unpub. data). All *C*. *gattii* isolates from these patients in Washington belong to the VGIIa genotype, as indicated by RFLP (M. Morshed and L. Hoang, unpub. data) and MLST (*31*). Three of these 10 patients had no travel history to Vancouver Island or other *C. gattii* disease–endemic areas. In addition, 1 Washington resident with sarcoidosis received a diagnosis of *C*. *gattii* disease in Oregon; the isolate from this patient belonged to a novel VGII genotype, VGIIc (*31*). Washington State now considers *C*. *gattii* infection notifiable as a rare disease of public health significance (www.doh.wa.gov/notify/nc/cryptococcus.htm).

Cases of *C*. *gattii* disease have been identified in Oregon since 2004. During 2004–2005, two cases of culture-confirmed *C*. *gattii* infection were recorded in the United States. These case-patients did not report exposure to Vancouver Island or other *C. gattii*–endemic areas. Isolates from these case-patients (both Oregon residents) were VGII, but they were genetically distinct from Vancouver Island VGII isolates and their relationship to the outbreak is unknown (*27*). The current count is 19 confirmed cases, as of May 2009 (S. West and K.A. Marr, unpub. data). As with case-patients seen in British Columbia and Washington, many of these patients had pulmonary manifestations. A review of the travel history of these patients suggests in-state acquisition, although with the currently considered incubation period of 6 weeks to 11 months for *C. gattii* disease (*27*,*28*), out-of-state acquisition cannot be ruled out. Additional cases are suspected because of increased reports of cryptococcal disease documented in otherwise healthy persons (K.A. Marr, unpub. data). However, the current epidemiologic understanding of the extent and spread of *C*. *gattii* disease in Washington and Oregon is incomplete because of the lack of culture isolation and strain identification in clinical microbiology laboratories.

## Veterinary Cases in the Pacific Northwest

Cryptococcal disease in animals is often characterized by upper respiratory symptoms, subcutaneous nodules, pneumonia, central nervous system or ocular disorders, lymphadenopathy, or subcutaneous nodules (*2*,*24*,*32*). *C*. *gattii* infection was recognized in wild and domestic animals in British Columbia and Washington before human cases were detected, which reiterated the value of sentinel animal surveillance for emerging disease. To date, many species of animals, including dogs, cats, ferrets, porpoises, llamas, alpacas, birds, and a horse, have been confirmed to have *C*. *gattii* infections (*23*). During 2003–2005, *C*. *gattii* VGIIa infections were detected on the lower mainland of British Columbia (in 1 ferret, 1 llama, and 6 cats) (*27*) and in northern Washington (3 cats and 4 porpoises) (*27*; E. Byrnes, pers. comm.). From August 2006 through July 2008, *C*. *gattii* VGIIa was reported in 5 cats, 2 dogs, and 1 parrot in Whatcom, Island, Yakima, and Snohomish counties of Washington (R. Wohrle, pers. comm.). In Oregon, VGIIa *C*. *gattii* infection was found in 2007 in 1 cat in Lebanon, 1 dog in Salem, and 2 alpacas in McMinnville and Sherwood (*33*).

Identified risk factors for animals include disturbance of soil or vegetation caused by hiking, digging, logging, and construction. These activities can potentially increase aerial dispersal of the infectious particles and contact with soil and tree cuttings (*23*). The distribution of isolates recovered from human and animal sources and from the environment is shown in the Figure.

**Figure:**
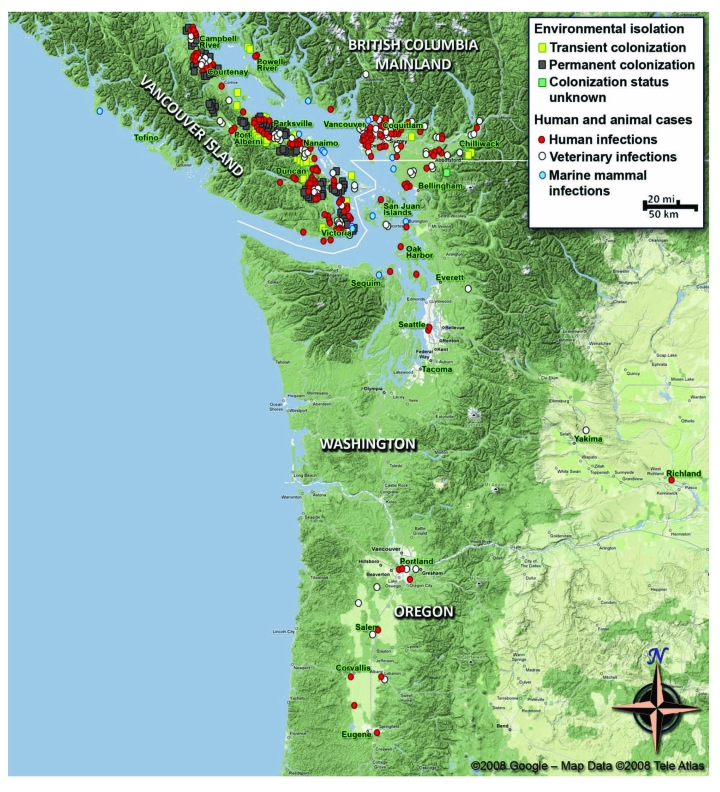
Map of the Pacific Northwest, comprising parts of British Columbia, Canada, and the states of Washington and Oregon in the United States, showing human and veterinary *Cryptococcus gattii* cases (including marine mammals) by place of residence or detection, and locations of environmental isolation of *C. gattii* during 1999–2008 (strain NIH444 [Seattle] or CBS7750 [San Francisco] not included). Data were collected from various state health departments and published reports referenced in the text. The map and icons have been used at a scale that shows gross geographic areas, effectively masking any personally identifiable patient locality information. Use of the map is courtesy of exclusive permission from Google Maps: ©2008 Google, map data ©2008 NAVTEQ.

## Ecologic Considerations

A possible association between *C*. *gattii* disease distribution and eucalyptus trees was observed in the early 1990s in Australia (*2*), and sporadically elsewhere (*34*,*35*). However, this association is not uniform (*11*), which indicates additional environmental sources. In British Columbia, *C*. *gattii* has been isolated from surfaces of >10 noneucalyptus tree species, soil, air, freshwater, and seawater within the Coastal Douglas Fir and Coastal Western Hemlock biogeoclimatic zones. These zones are characterized by warm, dry summers and mild, wet winters, a climate different from that traditionally associated with *C*. *gattii* (*23*). This finding suggests either a change in the ecology and distribution of this organism, or identification of a previously unrecognized niche (*23*,*27*).

From the beginning of the *C*. *gattii* epidemic on Vancouver Island and adjoining areas, all humans and animals with cryptococcal infection either lived within or traveled to the areas where *C*. *gattii* has been repeatedly and consistently isolated in subsequent studies (*23*,*27*). The Coastal Douglas Fir and Coastal Western Hemlock biogeoclimatic zones are located along the eastern edge of Vancouver Island, and extend into the southern Gulf Islands and lower mainland of British Columbia. Ecologic modeling of *C*. *gattii* in British Columbia has identified niche areas that are characterized by low-lying elevations (<770 m, average 100 m above sea level) and above freezing daily winter average temperatures (S. Mak, unpub. data). Similar climates with comparable temperature and rainfall extend further south into Washington and Oregon. Additionally, the San Juan Islands, Puget Sound, and the Willamette Valley contain plant diversity ecologically similar to that on Vancouver Island and in mainland British Columbia, lending support to the idea that *C*. *gattii* may have niche areas suitable for colonization in the broader Pacific Northwest (*27*).

Large-scale environmental sampling conducted from October 2001 through December 2005 in mainland British Columbia, the Gulf Islands, and Washington showed that 60 (3%) of 2,033 non-Vancouver Island environmental samples (air, water, soil, swabs of trees and other structures) were positive for *C*. *gattii* serotype B (58 VGIIa, 2 VGI) (*27*). *C*. *gattii* was consistently isolated from some areas. However, it was not found in the environment in other areas, such as the San Juan Islands (*36*), which suggests that environmental hot spots (zones of high concentration) of *C*. *gattii* may be found within the same broad ecologic niches. Some areas may have transient colonization, with initially positive sites yielding subsequent negative results (*23*).

Complicating the interpretation of epidemiologic data is the fact that the nature of the infectious propagule is currently unknown. Air sampling on Vancouver Island and in northern Washington has detected particles that are small enough to be either desiccated yeast cells or spores (*23*). *Cryptococcus* species are haploid yeasts that predominantly reproduce asexually (through mitosis and budding). However, they also possess a bipolar mating system, with mating types a or α. These mating types are capable of completing a sexual (meiotic) cycle that is either heterosexual (between a and α) or unisexual (nonclassical union between α and α or a and a; also known as monokaryotic fruiting) (*21*). This cycle can produce spores that can be up to 100-fold more infectious than yeast cells and thus might represent the infectious source (*37*). In tree hollows, *C*. *gattii* populations can exist equally as only α-mating type isolates or as a- and α-mating type isolates; both undergo recombination (*38*). This observation, taken together with the isolation of a diploid, homozygous α-mating type *C*. *gattii* strain from Vancouver Island, indicates that unique monokaryotic fruiting between α-mating type strains may be producing the infectious spores on Vancouver Island (*20*). Moreover, recent studies have shown that *C*. *gattii* is stimulated to complete its sexual cycle during an association with plants (*39*).

The origin or introduction of *C*. *gattii* strains into the Pacific Northwest and factors encouraging their emergence as disease agents remain a mystery. Accurate determination of origin from any particular locale is difficult because of global dispersal and isolate recombination (*19*). Several hypothesized disease movement models have been inconclusive. As discussed above, *C*. *gattii* VGIIa strains may have existed for the past 35 years in the Pacific Northwest, and changing conditions of climate, land use, or host susceptibility may have caused it to emerge at this time. An alternative hypothesis is that the VGIIa genotype is well adapted to the local biogeoclimatic conditions and enhanced with regards to virulence by virtue of the same-sex mating of the parents (*20*). Genetic studies putatively show that the dominant strain on Vancouver Island (VGIIa) originated in Australia, South America, or the Pacific Northwest. Conversely, the Vancouver Island minor genotype (VGIIb) is identical to fertile isolates from Australia and may well have originated on that continent (*20*,*40*).

## Issues and Questions

We will now attempt to identify several major unmet needs regarding *C*. *gattii* disease in the areas of public health, human and veterinary medicine, environmental science, and microbiologic or basic research. Although enhancing a multidisciplinary awareness of *C*. *gattii* infection will encourage prompt, accurate diagnosis of patients with compatible clinical symptoms, many clinical microbiology laboratories in the United States are not currently equipped to identify the organism to the species level. Most laboratories use antigen testing or histopathologic analysis and report all isolates simply as *C*. *neoformans*; few laboratories use biochemical tests that provide species level differentiation. This practice should be reconsidered, especially in laboratories servicing disease-endemic regions.

Although formal *C*. *gattii* surveillance has been ongoing in British Columbia since 2003, no routine surveillance program currently exists in the United States. Therefore, identified cases almost certainly represent an underestimate of the actual incidence of disease. Washington State has recently initiated a surveillance system for human cryptococcosis cases (www.doh.wa.gov/notify/nc/cryptococcus.htm). However, to gather sufficient information about the true incidence of this disease and to perform necessary clinical studies, a coordinated, systematic, regional approach to human and veterinary surveillance is needed.

Currently, the University of British Columbia and the British Columbia Centre for Disease Control hold a large collection of *C*. *gattii* isolates isolated from environmental, animal, and human sources. Additional central repositories of *C*. *gattii* strains and clinical databases would be useful for investigators in the field.

Animal models of *C gattii* disease are necessary for studying the pathogenicity of genetically diverse isolates and host immunity, as well as to answer more fundamental questions about the disease. Laboratory-based efforts should also focus on development of better diagnostic aids, such as a reliable system to isolate DNA from fixed tissue. Development of species-specific serologic tests would enable seroprevalence studies for a better understanding of epidemiology and disease mechanisms. Studies of soil persistence and propagation of the organism and those that address the nature and factors controlling the infectious propagule would be informative (*23*).

Antifungal drug susceptibility testing represents an area in need of improvement. Current susceptibility testing methods do not consider the unique in vitro growth conditions of cryptococci, and interpretation of results is difficult in the absence of clinical breakpoints.

Some standardization in the performance of molecular techniques for genomic research is required. Different genotyping methods have their own particular advantages and disadvantages, and the method of choice varies considerably among different laboratories. Random amplified polymorphic DNA analysis, RFLP, and amplified fragment length polymorphism analysis provide a lower resolution starting point, but may not be sufficient to rigorously establish strain identities, for which MLST approaches are necessary. The appropriate method is obviously dependent on the desired level of discrimination of the strains. However, because the organism designations may vary with different methods, common nomenclature should be standardized across molecular methods. Finally, further genome efforts should be encouraged; information comparing the genetic makeup of VG (I–IV) lineages might hold the key to understanding the differences in niches, virulence, mating, and the ability to cause an outbreak.

From a long-term public health perspective, it is important to gain an understanding of how *C*. *gattii* disease was introduced and spread within the Pacific Northwest, including the possible role of global climate change, deforestation and increased land use, as well as factors related to the biology of the organism and changing host susceptibility. This knowledge, acquired through environmental and genomic research studies, would help identify high-risk groups or regions, and provide information on risk-reduction and improvement of diagnosis and treatment.

## Conclusions

*C*. *gattii* has become endemic to the Pacific Northwest. Efforts to understand the emergence on Vancouver Island and mainland British Columbia have generated valuable information. However, many questions remain unanswered. The Centers for Disease Control and Prevention (Atlanta, GA, USA) is currently working in conjunction with state public health departments to enable clinical (human and veterinary) surveillance. Additional organized national and international efforts are needed. As the first step, *C*. *gattii* should be recognized as an emerging infection in the United States and declared a priority pathogen in the eyes of funding agencies.
